# Grape Seed Oil Attenuates Myocardial Fibrosis by Inhibiting the PI3K/AKT Signaling Pathway

**DOI:** 10.3390/foods15071182

**Published:** 2026-04-01

**Authors:** Ruixin Jing, Pilian Niu, Ruofen Wang, Guannan Zhang, Mingsheng Bai

**Affiliations:** 1 Life Science School, Ningxia University, Yinchuan 750021, China; 15296926623@163.com (R.J.); 15291029266@163.com (P.N.); wrf660907@163.com (R.W.); zgn14709551182@163.com (G.Z.); 2 Key Laboratory of the Ministry of Education for the Conservation and Utilization of Special Biological Resources of Western China, Ningxia University, Yinchuan 750021, China; 3 School of Life Science and Technology, Inner Mongolia University of Science & Technology, Baotou 014000, China

**Keywords:** network pharmacology, myocardial fibrosis, grape seed oil, molecular docking, cardiac fibroblasts, α-smooth muscle actin

## Abstract

**Background**: Myocardial fibrosis (MF) results from excessive collagen deposition in the cardiac interstitium, causing structural and functional cardiac impairments that underlie multiple cardiovascular diseases. Grape seed oil (GSO), rich in various bioactive fatty acids, demonstrates established cardiovascular benefits, yet its potential mechanisms against MF remain incompletely elucidated. This study was designed to investigate the inhibitory effects of bioactive components from GSO on TGF-β1-induced fibrosis in cardiac fibroblasts (CFs) and to elucidate the underlying molecular mechanisms. **Methods**: GSO was obtained using supercritical CO_2_ extraction technology. Initially, the anti-fibrotic activity of GSO was evaluated in vitro: a fibrosis model was established by inducing cardiac fibroblasts with TGF-β1 (10 ng/mL for 48 h), followed by treatment with 20% (*v/v*) GSO. Subsequently, the bioactive constituents of GSO were identified by Gas Chromatography-Mass Spectrometry (GC-MS). Network pharmacology approaches were employed to predict its potential therapeutic targets and associated signaling pathways. Molecular docking simulations were then performed to validate the binding interactions between the key bioactive components and the core targets obtained from enrichment analysis. Finally, the predicted core pathway was experimentally verified by Western blot analysis. **Results**: In vitro experiments demonstrated that 20% GSO treatment significantly downregulated TGF-β1-induced fibrotic markers at both transcriptional (*MMP9*, *MMP2*, *Col1a1*) and protein (TGF, Col I/III, α-SMA) levels (*p* < 0.01). GC-MS analysis identified nine fatty acids in GSO, including palmitic acid and linolenic acid. Network pharmacology revealed interactions between these compounds and 357 myocardial fibrosis-related targets. Molecular docking confirmed strong binding affinities (below −5.0 kcal/mol) of key components (heptadecanoic acid, palmitic acid) to core targets (MMP-9, PTGS2, MAPK3). Western blot analysis further verified that GSO significantly inhibited the expression of PI3K-AKT pathway-related proteins (*p* < 0.01). **Conclusions**: The fatty acids in GSO (linolenic acid, palmitic acid) attenuate myocardial fibrosis by inhibiting the PI3K/AKT signaling pathway and downregulating key fibrotic markers. These findings establish a novel theoretical foundation for the treatment of myocardial fibrosis and highlight the potential value of grape industry byproducts in cardiovascular therapeutics.

## 1. Introduction

Myocardial fibrosis (MF) is a pathological process characterized by excessive activation of cardiac fibroblasts in response to chronic injury or pathological stimuli, leading to aberrant deposition of extracellular matrix (ECM) components, including collagen and fibronectin [1]. This maladaptive remodeling disrupts myocardial architecture and serves as a common pathological substrate for the progression of various cardiovascular diseases (CVD) [2]. Furthermore, MF-induced disruption of cardiac electrophysiological conduction pathways directly promotes arrhythmias, which consequently elevate the risks of sudden cardiac death and heart failure [3]. Therefore, developing effective MF treatments represents a critical therapeutic strategy for preventing and managing CVD.

Traditional Chinese Medicine (TCM) has significant advantages in the treatment of chronic conditions such as heart disease and myocardial fibrosis [4]. Furthermore, essential oils extracted from medicinal plants exhibit favorable safety and low toxicity. Numerous studies have confirmed the significant efficacy of volatile oil components from Traditional Chinese Medicine in treating cardiovascular diseases (CVD) and preventing organ fibrosis [5,6,7]. Therefore, the discovery of the volatile oils derived from medicine–food homology plants in Traditional Chinese Medicine for the treatment of MF could be an effective approach. In line with this focus on bioactive lipids from natural sources, the present study investigates grape seed oil (GSO), a representative and studied source of nutritious and potentially therapeutic lipids.

Grape seeds, which are byproducts of winemaking derived from *Vitis vinifera* L., are processed using various extraction methods—such as cold pressing and supercritical CO_2_ extraction—to obtain grape seed oil (GSO) [8]. This oil is abundant in a diverse array of bio-active compounds [9]. The majority of these compounds are fatty acids, including linoleic acid and oleic acid [10], along with phenolic compounds and phytosterols [11]. These constituents are recognized for their beneficial effects, which include promoting vasodilation, exhibiting antioxidant properties, and reducing cholesterol levels [12]. Recent studies have indicated that its active constituents possess therapeutic properties for CVD. Franca Marangoni et al. found that linoleic acid (LA) can significantly reduce the risk of cardiovascular diseases [13]. Meanwhile, Garavaglia et al. demonstrated that the active compounds in grape seed oil, such as linoleic acid and tocopherols, possess antioxidant and cardioprotective effects [14]. Network pharmacology has emerged as a powerful approach in modern pharmacological research, enabling systematic exploration of drug–disease interactions through integrative analysis of multi-source databases. This methodology is particularly suitable for investigating cardiovascular diseases (CVDs), given their complex pathogenesis involving dysregulation of multiple proteins and signaling pathways [15]. Furthermore, it is suitable for the multi-component nature of herbal volatile oils, which often exhibit polypharmacological effects through synergistic.

Therefore, this study utilized gas chromatography-mass spectrometry (GC–MS) to characterize the principal chemical constituents of GSO. Using network pharmacology, we sought to identify its key bioactive compounds along with their potential targets and pathways implicated in the treatment of myocardial fibrosis (MF). Furthermore, the putative targets and associated signaling pathways through which GSO exerts its anti-fibrotic effects were experimentally validated using molecular docking and in vitro cellular models.

## 2. Materials and Methods

### 2.1. Preparation of GSO

Grape seed oil (GSO) is extracted from grape seeds sourced from the eastern foothills of the Helan Mountain in Ningxia using supercritical carbon dioxide extraction technology [16]. The detailed extraction parameters and the specific batch information of the grape seed oil are provided in the Supplementary Methods. The preparation protocol for the GSO stock solution is detailed in the Supplementary Methods.

### 2.2. Cell Experiments

#### 2.2.1. Cell Culture

CFs were isolated from the heart tissue of 1–3 day neonatal C57BL/6 mice. The mice were euthanized, and the cardiac apex was excised. The tissue was rinsed three times with cold PBS containing antibiotics and then minced into approximately 1 mm^3^ pieces. Digestion was performed using trypsin at 37 °C. Following digestion, the cells were collected by centrifugation and cultured in DMEM supplemented with 10% FBS and 1% antibiotics. After 1.5 h of culture, the supernatant containing non-adherent cells was discarded. The adherent cells were then maintained in fresh DMEM culture medium. The CFs cell culture and MF model establishment were conducted based on previous research from the research group [17]. After treating CF cells with TGF-β1 at a concentration of 10 ng/mL for 48 h, a stable cardiac fibrosis cell model was established [18,19]. All experiments were performed using cells at passages 3 to 8 to ensure consistency and viability.

#### 2.2.2. Cell Viability Analysis

Cell viability was assessed using CCK-8 assay. CFs in good condition were seeded at a density of 1 × 10^4^ cells per well in a 96-well plate, after 12 h of incubation. The cells were treated with the respective conditions for 48 h. Then, 10 µL of CCK-8 reagent was added to each well. To account for the effect of the solvent, a control group containing the corresponding concentration of Tween-80 (used in GSO stock solution) was also included. The experimental groups were as follows: blank control group (without cells), a control group (cells without treatment), a model group (10 ng/mL TGF-β1), Tween-80 group and a model group treated with GSO. After a 2 h incubation, the absorbance (A) at 450 nm was measured. Cell viability was calculated using the following formula: Cell viability = (A treated − A blank )/(A control − A blank).

#### 2.2.3. Immunofluorescence

CFs cells were seeded at a density of 1 × 10^5^ cells in a 6-well plate containing sterile glass slides. After 48 h of incubation according to the group assignments (control group, model group, GSO treatment group), the cells were washed with PBS and fixed with 4% paraformaldehyde for 10 min. The cells were then permeabilized with 0.1% Triton X-100 for 10 min and blocked with 5% BSA for 60 min. Subsequently, the cells were incubated overnight at 4 °C with α-SMA antibody (1:500). Afterward, a rabbit green fluorescent secondary antibody (1:500) was applied in the dark for 60 min, followed by DAPI staining for 10 min. The slides were then mounted and observed under a fluorescence microscope for imaging.

#### 2.2.4. Quantitative Real-Time PCR

RT-qPCR was used to detect the expression levels of MF-related genes *Vim*, *Acta2*, *Col1a1*, *MMP-9*, and *MMP-2* after 20% GSO treatment. After seeding CFs cells in a 6-well plate for 12 h, the cells were grouped and treated for 48 h. Then, TRIzol reagent (15596026, Invitrogen, Carlsbad, CA, USA) was used to collect the cells. Total RNA was extracted according to the method previously reported by our research group, and then reverse transcribed into cDNA following the instructions of the kit. Real-time quantitative PCR was performed using ChamQ Universal SYBR qPCR Master Mix (R711, Vazyme, Nanjing, China) according to the manufacturer’s instructions, with the reaction conditions based on our group’s previous reports. GAPDH was used as the internal control for quantification, and the primer sequences for other genes are listed in Table 1. The resulting data were recorded as Ct values, and RNA expression was further analyzed using the 2^−ΔΔCt^ method.

#### 2.2.5. Western Blot

CFs cells were seeded at a density of 1 × 10^5^ cells per well in a 6-well plate and treated with drugs for 48 h. After treatment, the cells were washed three times with PBS, followed by the addition of RIPA cell lysis buffer (KGP250, KeyGEN, Nanjin, China). The cells were lysed for 30 min, and the total protein was collected using a cell scraper. After quantification with a BCA assay kit, the samples were boiled in water at 100 °C for 10 min to denature the proteins, followed by SDS-PAGE electrophoresis. After electrophoresis, the proteins were transferred to a PVDF membrane. The membrane was blocked with 1% non-fat milk at room temperature for 1 h, and then incubated with the primary antibody overnight at 4 °C. The next day, the membrane was incubated with the secondary antibody for 60 min. ECL chemiluminescent substrate was applied to the membrane, and imaging was performed using a chemiluminescent imaging system. Protein bands were scanned using ImageJ (1.51J8) software, and the ratio of the target protein to β-actin was calculated for statistical analysis.

### 2.3. Compound Identification of GSO

The fatty acid composition of grape seed oil was analyzed by gas chromatography-mass spectrometry (GC-MS). The sample was subjected to methylation using a potassium hydroxide-methanol solution prior to analysis. Detailed derivatization procedures are provided in the Supplementary Materials. GC-MS analysis was performed on an Agilent 8860 GC system coupled with a 5977B mass spectrometer (Agilent Technologies, Santa Clara, CA, USA). Separation was achieved on an HP-5ms capillary column (30 m × 0.25 mm × 0.25 μm). The carrier gas was helium (He) with a flow rate of 1.0 mL/min. A sample volume of 1 μL was injected in split mode (split ratio 20:1) with the injector temperature set at 280 °C. The oven temperature was programmed as follows: initial temperature 140 °C held for 3 min, then ramped to 280 °C at 6 °C/min, and finally held at 280 °C for 5 min. The mass spectrometer was operated in electron impact (EI) ionization mode at 70 eV. The ion source temperature was 280 °C. Data were acquired in full scan mode over a mass range of *m*/*z* 30–550. Compounds were identified by comparison with the NIST 2014 mass spectral library.

### 2.4. Network Pharmacology Analysis

#### 2.4.1. Prediction of Active Ingredients and Targets of GSO

The active components of GSO were identified based on the results of GC-MS analysis. We queried the PubChem database for the structural formulas of the volatile active compounds identified by GC-MS, then collected and integrated the target proteins for each active component using databases such as SwissTargetPrediction and TCMSP.

#### 2.4.2. Screening of Targets for GSO Against Myocardial Fibrosis

To identify the targets associated with MF relevant to GSO, we sourced disease-related target information from several databases, including GeneCards, OMIM, and DrugBank. Subsequently, we utilized the Venn online platform to analyze and obtain the overlapping targets that indicate the potential efficacy of GSO in combating myocardial fibrosis. This integrative approach allows for a clearer understanding of the molecular interactions and therapeutic potential of GSO in the context of MF.

#### 2.4.3. Network Enrichment and Analysis

The identified targets of GSO against myocardial fibrosis (MF) were imported into the STRING database to construct a protein–protein interaction (PPI) network. Following the generation of the PPI network, we utilized the CytoNCA plugin to calculate the degree centrality of each protein, enabling us to identify and visualize the core targets within the network. Subsequently, we performed Gene Ontology (GO) and Kyoto Encyclopedia of Genes and Genomes (KEGG) enrichment analyses on the core targets. A significance threshold of *p* < 0.001 was applied to filter the results. The findings were then visualized using R (4.5.1) software, allowing for a comprehensive interpretation of the biological processes and pathways associated with the core targets.

### 2.5. Molecular Docking

To study the combining capacity between the Top10 key targets and GSO marker components, the AutoDock Vina (1.5.6) software was used to perform molecular docking. The 3D structures of the active components of GSO were obtained from the PubChem database, while the 3D structures of proteins were sourced from the PBD database. Molecular docking of the ligand and receptor was performed using AutoDock Vina (1.5.6) software.

### 2.6. Statistical Analysis

The raw data from GC-MS were processed using Microsoft Excel, recording the names of all samples, retention times, peak intensities, and mass-to-charge ratios. In the cytological experiments, each experiment was performed in triplicate, and all experiments were performed with a minimum of three independent biological replicates (n ≥ 3). Data are presented as the mean ± standard deviation (SD). Statistical analysis was performed using GraphPad Prism (9.5.0) software. Differences between multiple groups were analyzed by one-way analysis of variance followed by Tukey’s post hoc test for pairwise comparisons. A *p*-value of less than 0.05 (*p* < 0.05) was considered statistically significant.

## 3. Results

### 3.1. Cellular Experiments for Validation

#### 3.1.1. The Effect of GSO on the Viability of CFs

**Figure 1:**
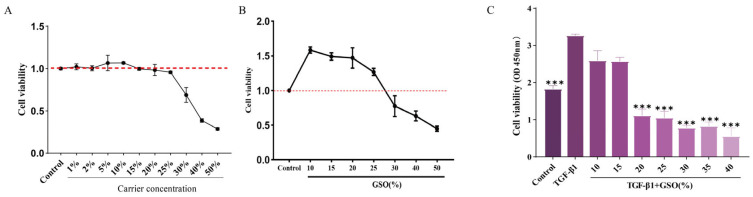
Effect of different treatments on the vitality of CFs. (**A**) Cytotoxicity of Tween-80 (solvent control). (**B**) Cytotoxicity of grape seed oil (GSO). (**C**) Effect of GSO on TGF-β1-induced alterations in cell viability. (Data are presented as mean ± standard deviation (SD) (n = 3). *** *p* < 0.001 compared to the TGF-β1 group).

The cell viability of each group was assessed using the CCK-8 assay. To assess the safety of the vehicle itself, we first evaluated the cytotoxicity of the Tween 80-based nano-emulsion (without GSO). The results indicated that the vehicle alone at concentrations ranging from 1% to 25% (*v/v*) did not exhibit significant cytotoxicity towards CFs (*p* < 0.05, Figure 1A). The results indicated that there was no significant change in the viability of CFs treated with 10% to 25% GSO compared to the control group. However, treatment with GSO concentrations greater than 30% led to a significant decrease in CF viability (*p* < 0.05), and cell viability further declined with increasing concentrations (*p* < 0.05, Figure 1B). Additionally, following TGF-β1 stimulation, the model group exhibited a significant increase in cell proliferation compared to the control group (*p* < 0.05). In contrast, the TGF-β1 + 20% GSO group showed a significant decrease in CF proliferation (*p* < 0.05, Figure 1C). Therefore, a concentration of 20% GSO was selected for subsequent experiments.

#### 3.1.2. The Effect of GSO on CFs

**Figure 2:**
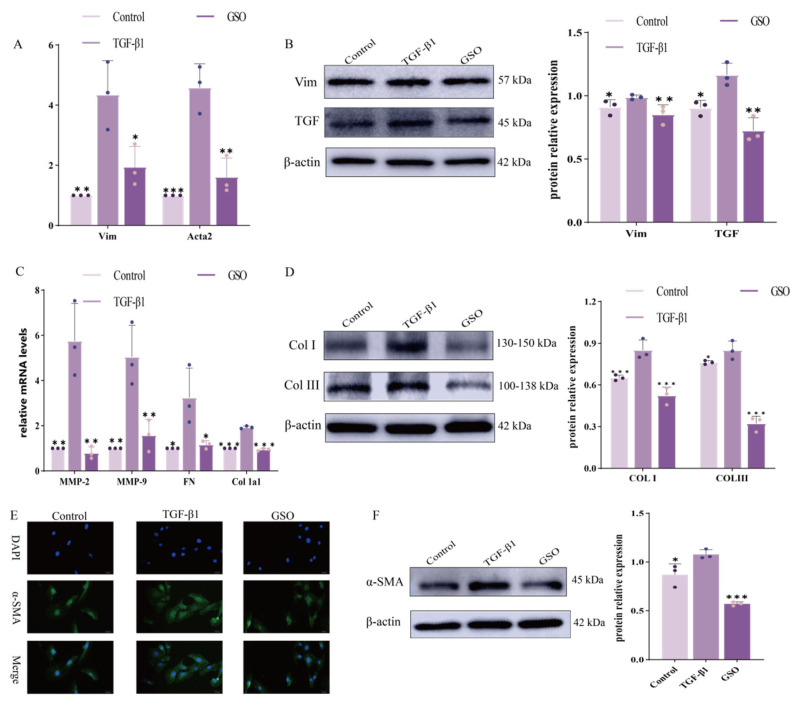
Effect of GSO on TGF–β1 stimulation of CFs. (**A**) *Acta2* and *Vim* mRNA expression of in CFs. (**B**) The expression of TGF and Vim proteins in TGF–β1–stimulated CFs. (**C**) *Col1a1, FN, MMP2* and *MMP9* mRNA levels in TGF–β1–stimulated CFs. (**D**) Col I and Col III proteins in TGF–β1–stimulated CFs. (**E**) α–SMA protein in GSO-treated CFs using immunofluorescence. (**F**) Western blotting revealed that GSO treatment reversed the expression of TGF-β1-stimulated α–SMA protein. (* *p* < 0.05, ** *p* < 0.01, *** *p* < 0.001 compared to the TGF–β1 group).

After stimulation with TGF-β1, the expression of mRNA for *MMP9, MMP2*, *fibronectin (FN)*, and *Col1a1* in CFs significantly increased; however, treatment with GSO reversed this trend (Figure 2A). Similarly, GSO treatment inhibited the expression of Col I and Col III proteins in CFs stimulated by TGF-β1 (Figure 2B). Furthermore, GSO treatment also reduced the mRNA levels of *Acta2* and *Vim* in TGF-β1-stimulated CFs (Figure 2C) and decreased the expression of fibrosis-related proteins such as Vim, TGF, and α-SMA (Figure 2D). Protein blotting results indicated that treatment with 20% GSO reversed the expression of α-SMA protein in CFs stimulated by TGF-β1 (Figure 2F). Subsequently, our immunofluorescence analysis confirmed these findings, showing that the fluorescence intensity of α-SMA protein in GSO-treated CFs was significantly lower than that in CFs stimulated with TGF-β1 (Figure 2E).

### 3.2. Determination of the Composition of Grape Seed Oil

**Figure 3:**
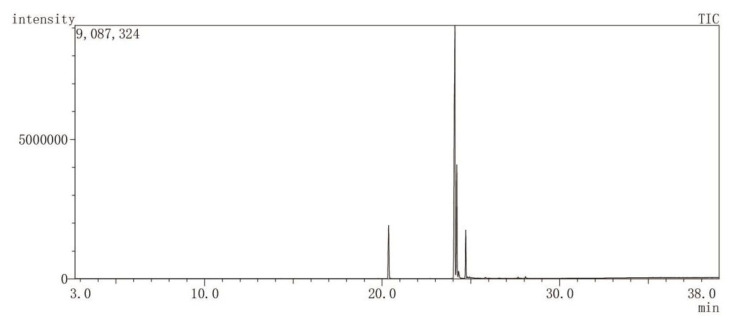
Total Ion Chromatogram of grape seed oil.

The chemical composition of GSO is summarized in Table 2, with the total ion chromatogram presented in Figure 3. Nine fatty acids were identified, accounting for 99.04% of the total peak area in GSO volatile oil. The major constituents included Hexadecanoic acid (10.06%) and Octadecadienoic acid (59.49%), with Octadecadienoic acid being the most abundant component.

### 3.3. Network Pharmacology Predicts the Mechanism of GSO in the Treatment of MF

#### 3.3.1. Relevant Targets of CGA and Myocardial Fibrosis

**Figure 4:**
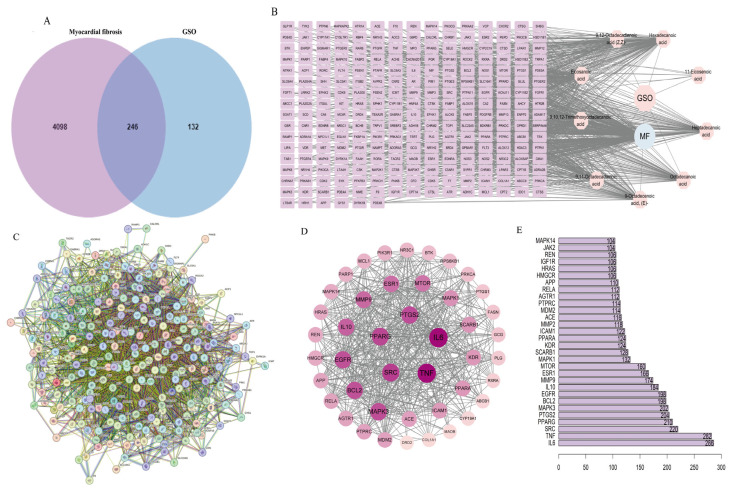
Network pharmacology-based prediction of core targets for grape seed oil against myocardial fibrosis. (**A**) Identification of overlapping drug-disease targets (Venn diagram). (**B**) GSO-MF-target interaction network. (**C**) Construction and analysis of the PPI network for intersecting targets. (**D**) Core target subnetwork based on topological analysis. (**E**) Ranking of key targets by network centrality (degree value).

A total of 4344 targets associated with myocardial fibrosis (MF) were identified from the GeneCards, DrugBank, OMIM, and DisGeNET databases. The search was conducted using the term “myocardial fibrosis,” with a significance threshold set at *p* < 0.01. Based on the chemical composition data of GSO obtained from GC-MS, drug targets were collected using the TCMSP and Swiss Target Prediction databases. After removing duplicate entries and standardizing the data through the UniProt database, a total of 378 potential targets were identified. A Venn diagram was constructed to identify the common targets between GSO targets and genes associated with myocardial fibrosis, revealing a total of 246 shared targets (Figure 4A).

#### 3.3.2. Construction of the “GSO-Components-Targets” Network and Screening of Core Targets

Using Cytoscape 3.9.0 software, a network diagram of active components and intersecting targets was constructed (Figure 4B). The potential common targets of the 246 shared GSO components were transferred to the STRING platform, resulting in a protein–protein interaction network (Figure 4C). After removing single-node genes, the protein interaction data were refined. Subsequently, the CytoNCA plugin was employed to identify the top 10 core targets through comprehensive topological analysis incorporating three centrality metrics: Degree, Betweenness Centrality, and Closeness Centrality (Figure 4D), with the degree values displayed in Figure 4E (The specific values of the topological parameters (Degree, Betweenness Centrality, and Closeness Centrality) calculated by CytoNCA for all intersecting targets are provided in Supplementary Table S1).

#### 3.3.3. GO and KEGG Enrichment Analysis of Core Targets

**Figure 5:**
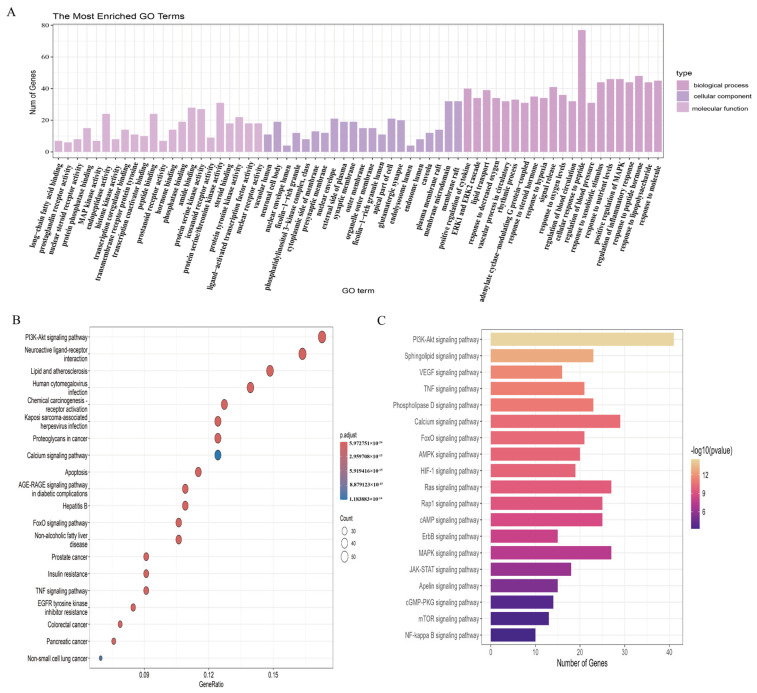
Functional annotation and signaling pathway enrichment analysis of targets. (**A**) Gene Ontology (GO) enrichment analysis (biological process, cellular component, molecular function). (**B**) KEGG pathway enrichment analysis (bubble chart). (**C**) Significance ranking of key signaling pathways (bar chart).

GO enrichment analysis was performed on the 246 GSO–MF cross–targets. The analysis covered three aspects: Biological Process (BP), Cellular Component (CC), and Molecular Function (MF), resulting in a total of 2346 BPs, 82 CCs, and 235 MFs. The top 20 terms based on “Count” were selected to create a histogram (Figure 5A). Subsequently, KEGG enrichment analysis was conducted on the 246 targets, revealing their complex associations with signaling cascades, including the PI3K–AKT signaling pathway, neuroactive ligand–receptor interactions, and pathways related to lipids and atherosclerosis (Figure 5B). The top 20 signaling pathways were ranked (Figure 5C), with PI3K–AKT identified as the most significant pathway. To investigate the specific effects of GSO on MF within the PI3K–AKT signaling pathway, we examined relevant targets associated with this pathway, including *PI3K, AKT,* and *MMP9*.

### 3.4. Molecular Docking Evaluation

**Figure 6:**
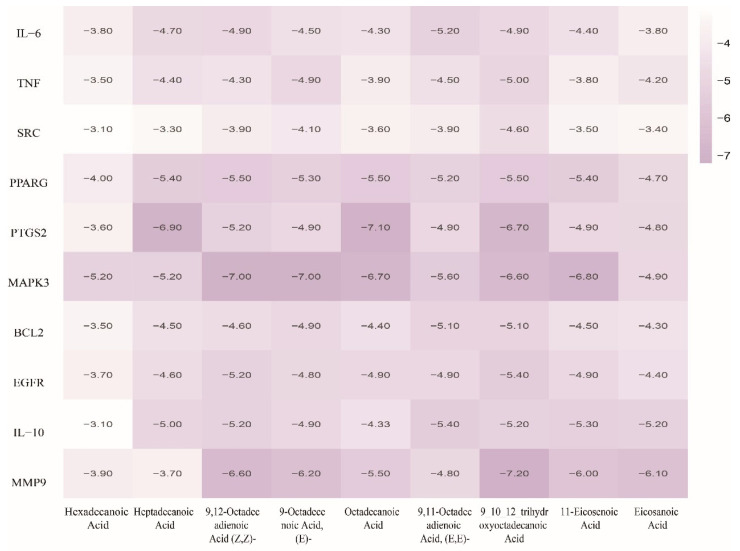
Heatmap of binding energies for grape seed oil fatty acids against core targets (Stronger binding is indicated by deeper purplish-red).

Molecular docking revealed strong binding affinities (<−5 kcal/mol) between GSO’s nine primary fatty acids and the top 10 core targets: Heptadecanoic acid–PTGS2 (COX-2), Octadecanoic acid–MMP9, and Tetradecanoic/Hexadecanoic acid–MAPK3 (ERK1) indicated a favorable binding affinity, suggesting that the active ingredients effectively interact with targets (Figure 6). The specific binding energy data from the molecular docking analysis are provided in the Supplementary Materials.

### 3.5. The Effect of GSO on the Expression Levels of Key Proteins in the PI3K–AKT Pathway

**Figure 7:**
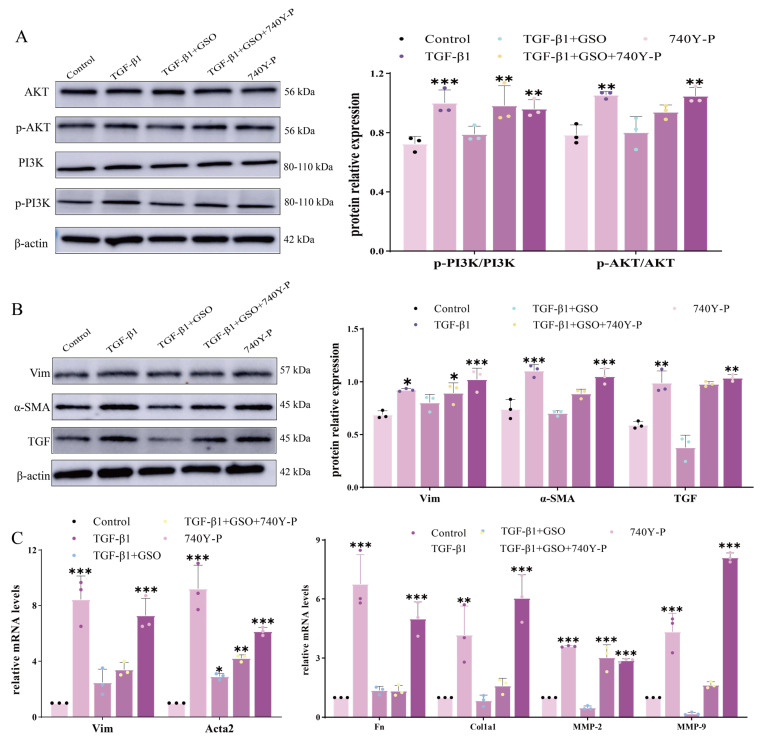
Effect of GSO on PI3K–Akt pathway protein expression. (**A**) Effects of GSO and its co–treatment with the PI3K activator 740Y–P on the activation of the PI3K/Akt signaling pathway in cardiac fibroblasts (CFs). (**B**) Effects of GSO and its co-treatment with 740Y–P on the expression of fibrotic marker proteins. (**C**) Effects of GSO and its co-treatment with 740Y-P on the mRNA expression levels of fibrosis–related genes. Data are presented as mean ± standard deviation (SD) (n = 3). * *p* < 0.05, ** *p* < 0.01, *** *p* < 0.001 compared to the Control group.

Based on the results of GO and KEGG enrichment analyses of core targets, it was found that the PI3K–AKT signaling pathway is associated with the MF effects of GSO. To validate the involvement of the PI3K–AKT pathway in the anti-fibrotic action of GSO, an experiment was performed using the PI3K–specific activator 740Y–P. As shown in Figure 7A, immunoblotting analysis revealed that TGF–β1 stimulation significantly increased the phosphorylation levels of both PI3K and AKT in CFs compared to the control. Treatment with GSO effectively reversed this TGF–β1–induced activation. Crucially, co–treatment with 740Y–P largely restored the phosphorylation levels of PI3K and AKT that were suppressed by GSO, indicating that pharmacological activation of PI3K can counteract the inhibitory effect of GSO on this pathway. Consistent with the upstream signaling changes, the expression of key fibrotic marker proteins was modulated in parallel. TGF–β1 stimulation markedly upregulated the protein levels of Vimentin, α-SMA, and TGF, which were significantly suppressed by GSO treatment (Figure 7B). Notably, the addition of 740Y–P partially but significantly reversed the GSO–mediated downregulation of these fibrotic proteins. At the transcriptional level, qPCR analysis demonstrated a coherent pattern (Figure 7C). TGF-β1 induced a substantial increase in the mRNA levels of fibrotic genes, including *Vim*, *Acta2, Fn*, *Col1a1*, *MMP2*, and *MMP9*. GSO treatment significantly attenuated these increases. Furthermore, the co-administration of 740Y–P with GSO reversed the inhibitory effect of GSO. Collectively, these data demonstrate that the activation of the PI3K–AKT pathway by 740Y–P can antagonize the suppressive effects of GSO on both the activity of this pathway and the expression of downstream fibrotic markers at both protein and gene levels. This experiment provides that GSO exerts its anti-fibrotic effect through the inhibition of the PI3K–AKT signaling pathway.

## 4. Discussion

MF is a common pathological feature of various cardiac diseases, including heart failure and coronary artery disease, and is primarily associated with the occurrence and prognosis of adverse cardiovascular conditions. Furthermore, studies have shown that MF is closely related to cardiac fibroblasts [20]. Under normal conditions, cardiac fibroblasts continuously remodel the cardiac microenvironment by degrading and depositing collagen and extracellular matrix (ECM). However, in response to pathological injury, the prolonged activation of specific signaling pathways in fibroblasts can lead to excessive ECM deposition and scar tissue formation, thereby exacerbating the progression of MF and contributing to the development of cardiac diseases [21]. Therefore, effective and timely management of myocardial fibrosis is crucial for the prevention and treatment of cardiac diseases.

Grape seeds are generated in large quantities as by-products of the global wine industry, presenting a significant opportunity for waste valorization. Research has shown that grape seeds contain an oil content of 10–17%, of which 80% consists of unsaturated fatty acids, indicating a high nutritional value [22]. Meanwhile, the literature reports that grape seed oil (GSO) possesses antioxidant properties. Excessive reactive oxygen species (ROS) in the body can lead to oxidative stress, which is associated with cancer, type II diabetes, pulmonary diseases, cardiovascular diseases, and degenerative disorders [23]. GSO has been demonstrated to effectively scavenge reactive oxygen species (ROS) and inhibit lipid oxidation [24], and reduce oxidized LDL levels [25], confirming its potential cardiovascular protective effects. However, the underlying mechanisms of its action remain unclear. To gain a comprehensive understanding of the mechanisms by which GSO treats myocardial fibrosis (MF), this study employs GC-MS, network pharmacology, and in vitro cellular assays to investigate the therapeutic effects of GSO on MF.

Through gas chromatography-mass spectrometry (GC–MS) analysis, we identified that the primary components of grape seed oil are fatty acids such as palmitic acid and linoleic acid, and identified a total of nine bioactive compounds. Network pharmacology–based analysis of the drug target database revealed that these nine compounds collectively interact with 378 targets, of which 246 are shared with targets related to myocardial fibrosis (MF), indicating that grape seed oil (GSO) may exert its therapeutic effects on MF through these 246 targets. Notably, IL-6 emerged as the highest–ranked key target. Research indicates that IL–6 is a classic pro-inflammatory cytokine involved in cardiovascular diseases such as ischemic heart disease and heart failure, where its acute phase response in cardiac tissue can promote thrombus formation [26]. Alter C demonstrated through the establishment of a myocardial infarction mouse model that IL–6 production by cardiac fibroblasts triggers a cytokine storm, exacerbating myocardial injury and driving atherosclerosis [27]. Our molecular docking analysis results indicate that the nine fatty acids demonstrate potential binding affinity to key targets such as IL–6 to varying degrees, with some fatty acids showing binding energies lower than −5 kcal/mol. These findings suggest that the fatty acid components in grape seed oil may exert anti-MF bioactivity by interacting with these core target proteins. It is important to know that different fatty acids can have different, even opposite, effects in the body. For example, linoleic acid is often linked to health benefits. However, in contrast to the beneficial actions of unsaturated fatty acids like linoleic acid, studies indicate that palmitic acid can promote inflammatory responses and contribute to disease progression under certain conditions. Notably, in the context of metabolic stress such as in nonalcoholic fatty liver disease (NAFLD), palmitic acid has been shown to induce lipid peroxidation and inflammation [28]. The strong anti–fibrotic effect we saw with the whole GSO extract likely comes from the mixed action of all its main components working together.

Subsequently, Gene Ontology (GO) and Kyoto Encyclopedia of Genes and Genomes (KEGG) enrichment analyses generated predictions, suggesting that grape seed oil (GSO) primarily exerts its anti–myocardial fibrosis (MF) effects through responses to oxidative stress and the PI3K–AKT signaling pathway. Based on these computational predictions, we prioritized the PI3K–AKT pathway for experimental validation. Under conditions of oxidative stress, reactive oxygen species (ROS) can activate the PI3K–AKT signaling pathway, which is closely associated with MF [29]. We performed a series of in vitro assays to test this hypothesis. Western blot analysis demonstrated a significant inhibition of PI3K and AKT phosphorylation following GSO treatment. Consistent with this finding, GSO administration resulted in the marked downregulation of key fibrotic mediators, including the transcriptional regulators *MMP9* and *MMP2* [30], as well as the marker proteins *ACTA2* and *Vim* [31]. Furthermore, GSO significantly reduced the expression of extracellular matrix components, specifically collagen and fibronectin [32,33], Vimentin (Vim), and Alpha smooth muscle actin (α-SMA) fibrosis-related marker proteins [34,35]. These collective results demonstrate that GSO ameliorates MF by suppressing the activation of the PI3K/AKT pathway and the subsequent expression of pro-fibrotic factors, thereby inhibiting cardiac fibroblast proliferation. The experimental data thus experimentally support the involvement of the PI3K/AKT pathway, which was a key prediction from the network pharmacology analysis. It should be noted that while our study focused on validating this central predicted pathway, the potential roles of other enriched pathways warrant further investigation in the future.

Currently, the integration of network pharmacology with GC-MS has emerged as an effective strategy for elucidating the mechanisms by which Traditional Chinese Medicine volatile oils exert therapeutic effects on diseases. For example, the volatile oils of *Cyperus rotundus* and *Perilla frutescens* have shown synergistic antidepressant effects, with studies utilizing GC–MS combined with network pharmacology and metabolomics revealing their mechanisms of action through the regulation of tryptophan and tyrosine biosynthesis and metabolism to inhibit the onset of depression [36]. Additionally, research on *Acorus tatarinowii* have revealed its potential anti–Alzheimer’s disease (AD) effects, with GC–MS and network pharmacology studies confirming that its volatile oils attenuate neuroinflammation via inhibition of the PI3K–AKT signaling pathway [37].

In conclusion, this study confirmed that grape seed oil can inhibit the key markers of myocardial fibrosis in vitro, and this effect may be achieved by regulating the PI3K-AKT signaling pathway. The above findings suggest that grape seed oil, as a dietary source rich in bioactive lipids, has the potential to intervene in the fibrotic process. However, this study has certain limitations. First, all validations were performed in cellular models; thus, the anti-fibrotic effects and underlying mechanisms of GSO remain to be further verified in animal models to better reflect physiological complexity. Second, while network pharmacology and molecular docking predicted the potential bioactive components in GSO, the anti-fibrotic activity of these individual compounds has not been experimentally confirmed. Future research should therefore focus on in vivo validation, isolation of the key active compound(s), and exploration of additional targets.

## Figures and Tables

**Table 1:** Sequence of primers.

Gene	Sequence of Primers (5′→3′)
*GAPDH*	F: GCAAATTCAACGGCACAGTCAAG
	R: TCGCTCCTGGAAGATGGTGATG
*Col 1a1*	F: AGGCGAACAAGGTGACAGAGG
	R: GGAGAACCAGGAGAACCAGGAG
*MMP-9*	F: AATAAAGACGACATAGACGGCATCC
	R: AGTTGTGGTGGTGGCTGGAG
*MMP-2*	F: CCATGCGGAAGCCAAGATGTG
	R: GGTTTCAGGGTCCAGGTCAGG
*Vim*	F: CTGCTGGAAGGCGAGGAGAG
	R: TCAACCGTCTTAATCAGGAGTGTTC
*Acta2*	F: ATGACCCAGATTATGTTTGAGACCT
	R: TCCAGAGTCCAGCACAATACCAG

**Table 2:** The main active ingredients of grape seed oil.

Number	tR/min	Ingredient	Peak Area/%	CAS	SI
1	20.368	Hexadecanoic acid, methyl ester	10.06	112-39-0	91
2	22.715	Heptadecanoic acid, methyl ester	0.03	1731-92-6	85
3	24.098	9,12-Octadecadienoic acid (Z, Z)-, methyl ester	59.49	112-63-0	92
4	24.208	9-Octadecenoic acid, methyl ester, (E)-	19.97	1937-62-8	91
5	24.709	Octadecanoic acid, methyl ester	7.37	112-61-8	97
6	25.205	9,11-Octadecadienoic acid, methyl ester, (E, E)-	0.04	13038-47-6	79
7	26.57	Octadecanoic acid, 9,10,12-trimethoxy-, methyl ester	0.1	55255-75-9	76
8	27.659	11-Eicosenoic acid, methyl ester	0.15	2390-09-2	81
9	28.078	Eicosanoic acid, methyl ester	0.22	1120-28-1	90

## Data Availability

Pubchem (https://pubchem.ncbi.nlm.nih.gov/), TCMSP (https://www.tcmsp-e.com), and Swiss TargetPrediction (http://www.Swisstargetprediction.Ch/) were used for GSO target screening. Screening of myocardial fibrosis targets was performed by GeneCards (https://www.genecards.org), OMIM (http:/www.omim.org), and DrugBank (https://www.drugbank.ca). STRING (https://string-db.org/) was used to draw the interaction network of disease-drug common target proteins. The core protein structure was downloaded in PDB (https://www.rcsb.org/) for molecular docking. Go (https://geneontology.org/) and KEGG (https://www.kegg.jp/) were used for gene function annotation and pathway enrichment analysis. The data generated in the present study may be requested from the corresponding authors.

## Supplementary Materials

The following supporting information can be downloaded at: https://www.mdpi.com/article/10.3390/foods15071182/s1, Table S1: Node topological properties (Degree, Betweenness Centrality, Closeness Centrality); Table S2: Binding Energies from Molecular Docking Between Core Target Proteins and Active Components; Table S3: Detailed Information of Western Blot Antibodies; Table S4: Extracted Ion Chromatograms for all nine identified fatty acid methyl esters.
