# Supplementary material for: Grape Seed Oil Attenuates Myocardial Fibrosis by Inhibiting the PI3K/AKT Signaling Pathway
Source: Foods. 2026 Apr 1;15(7):1182. doi: 10.3390/foods15071182 (PMC13072930; doi:10.3390/foods15071182)

Supplementary Method

1.CO_2 Extraction of Grape Seed Oil

The grape seeds used in this study were a winemaking by-product of the Cabernet Sauvignon variety, provided by Ningxia Huangkou Winery. The raw seeds were stored at -20℃. Pretreatment included washing with deionized water to remove impurities, followed by drying, mechanical decortication, grinding, and sieving through a 60-mesh sieve to obtain a homogeneous powder for subsequent extraction. Grape seed oil was extracted using supercritical CO_2 following an optimized method. Briefly, dried and crushed grape seed powder was loaded into the extraction vessel of a supercritical fluid extraction system. Extraction was performed under the following conditions: an extraction pressure of 28 MPa, a temperature of 45 ℃, and a flow rate of 700 L/h for a duration of 150 minutes. These conditions were established to maximize oil yield and quality[1].

Reference

[1] Xin ZHANG. Process and Device Design for the Supercritical CO2 Extraction of Grape Seed Oil [D]. Ningxia University, 2023. DOI: 10.27257/d.cnki.gnxhc.2023.001211.

2. Fatty Acid Methylation Procedure

For fatty acid methylation, approximately 0.2 g of grape seed oil was saponified by refluxing with 10 mL of 1 M sodium hydroxide in methanol solution for 1 h. After cooling to room temperature, concentrated sulfuric acid was added dropwise to acidify the mixture and catalyze the methylation. The reaction mixture was then refluxed for an additional 1 h. Following cooling, 2 mL of distilled water and 5 mL of n-hexane were added, and the mixture was vigorously shaken. After phase separation, the upper n-hexane layer containing the fatty

acid methyl esters (FAMEs) was collected for subsequent GC MS analysis.

3.Preparation of Grape Seed Oil Nanoemulsion

The grape seed oil (GSO) nanoemulsion was prepared using a high-energy emulsification method. Briefly, the oil phase was constituted by grape seed oil (100 μL) and the non-ionic surfactant Tween 80 (10 μL), which served as the emulsifier. The aqueous phase was phosphate-buffered saline (PBS, 890 μL). The two phases were mixed and then vigorously vortexed for 2 minutes to form a coarse pre-emulsion. This coarse emulsion was subsequently subjected to probe sonication on ice to prevent overheating. The sonication was performed at an amplitude of 40% for a total duration of 5 minutes, operating in a pulsed mode (5 seconds on, 5 seconds off). Final composition of Tween 80:GSO:PBS = 1:10:89 (v/v/v). For cell culture treatments, this stock solution was freshly diluted with complete culture medium to the desired final working concentrations (containing 20% v/v of the stock, equivalent to ~2.2% v/v GSO) and immediately applied to the cells.

4.Detailed Description on the Source, Reconstitution, and Bioactivity of TGF-β1

Human TGF-β1 was purchased from PeproTech (Catalog # AF-100-21C), and the specific batch used was Lot# 0918AF354. The lyophilized was reconstituted in PBS to prepare a 2 μg/mL stock solution, which was aliquoted and stored at -80°C to avoid repeated freeze-thaw cycles. For cell experiments, this stock solution was used to treat cardiac fibroblasts at a final concentration of 10 ng/mL for 48 hours to establish the in vitro fibrosis model. This modeling condition (10 ng/mL, 48h) was derived from our laboratory's previously published study[1-2], which systematically demonstrated that TGF-β1 treatment at this concentration and duration stably and significantly upregulates the expression of fibrotic

markers (α-SMA, Collagen I, Collagen III) via multiple techniques including Western Blot, qPCR, and immunofluorescence, successfully inducing a fibrotic phenotype. The present study directly adopted this thoroughly validated and established condition to ensure model reproducibility and stability. To reconfirm the efficacy of the specific batch used, we also performed a pilot experiment examining the expression of α-SMA and Collagen I by Western Blot, confirming that stimulation with 10 ng/mL TGF-β1 for 48 hours significantly induced their upregulation ($p < 0.01$).

[1] Pilian Niu, Xiangjun Zhang, Guannan Zhang, Ruixin Jing, Yarui Qiao, Xuezhang Zhou, Mingsheng Bai, Li Peng. A polysaccharide from Glycyrrhiza uralensis attenuates myocardial fibrosis via modulating the MAPK/PI3K/AKT signaling pathway[J]. International Journal of Biological Macromolecules, 2025, 286: 138207.

[2] Pilian Niu, Xiaoying Tan, Xuezhang Zhou, Xingbo Xu, Guannan Zhang, Li Peng, Mingsheng Bai. Novel polysaccharide identified from Cortinarius purpurascens demonstrated anti-fibrosis effects in cardiac fibroblasts[J]. Food Bioscience, 2023: 103157.

Supplementary Table S1. Node topological properties (Degree, Betweenness Centrality, Closeness Centrality) of the PPI network for myocardial fibrosis-related targets, analyzed by CytoNCA.

Gene	Degree	Betweenness Centrality	Closeness Centrality
IL6	143	5327.519	0.7096774
TNF	141	4881.7974	0.70348835
SRC	110	4821.5986	0.64705884
PPARG	105	3069.9639	0.6351706

PTGS2	102	2889.9888	0.6302083
MAPK3	101	1823.41	0.626943
BCL2	99	1715.9062	0.626943
EGFR	99	2193.784	0.626943
IL10	92	1366.887	0.6095718
MMP9	87	856.5246	0.605
ESR1	83	1120.8563	0.5990099
MTOR	80	1142.4617	0.5945946
MAPK1	66	695.41815	0.5707547
SCARB1	64	1023.4027	0.56674474
PPARA	62	1088.1458	0.5627907
KDR	62	448.6735	0.5680751
ICAM1	61	331.4483	0.5627907
MMP2	59	222.6179	0.56148493
ACE	58	854.8698	0.55632186
PTPRC	57	344.85236	0.5525114
MDM2	57	577.67706	0.5588915
RELA	56	398.8305	0.55632186
AGTR1	56	1361.6869	0.545045
APP	55	1165.8596	0.55760366
HRAS	53	425.82776	0.54875284
REN	53	652.5319	0.545045
IGF1R	53	176.76012	0.55125284
HMGCR	53	1081.0634	0.5401786
JAK2	52	181.29572	0.54875284
MAPK14	52	456.4335	0.541387
PIK3CA	50	108.9485	0.545045
KIT	50	197.95493	0.55
PARP1	49	286.24634	0.54260087
PIK3R1	48	630.83716	0.5377778
MAPK8	48	230.4904	0.5462754
PDGFRB	48	221.42648	0.5389755
MCL1	48	449.62598	0.53421634
PTPN11	47	224.61816	0.5401786
PGR	46	169.50465	0.5389755
NR3C1	46	584.2664	0.541387
RPS6KB1	43	356.4116	0.53421634
BTK	43	769.8263	0.5204301
NOS3	42	154.10922	0.5365854
PRKCA	42	705.66736	0.5330396
MPO	41	136.29456	0.5226782
SYK	39	69.84757	0.5272331
MAP2K1	39	82.58945	0.5272331
FASN	39	457.73044	0.51380044

AR	39	134.98946	0.5249458
PTGS1	39	458.409	0.5182013
GCG	39	371.38873	0.5295405
JAK1	38	50.64966	0.5116279
MET	38	53.974735	0.5182013
PLG	37	565.5866	0.51599145
PPARD	36	189.4424	0.517094
TERT	36	120.48867	0.5193133
NTRK1	36	140.29115	0.5182013
CTSB	36	209.2658	0.5148936
RXRA	35	304.9764	0.517094
PIK3CB	35	43.49042	0.49590164
ESR2	35	81.8122	0.526087
CDK2	35	157.05682	0.51599145
CTSS	34	134.2209	0.5204301
PIK3CG	33	149.09491	0.5226782
MMP3	33	48.18773	0.5073375
FGFR1	32	110.51257	0.5
PLA2G4A	32	234.46367	0.50840336
NOS2	32	84.45681	0.51054853
HNF4A	32	207.92934	0.5127119
ABCB1	32	654.59265	0.5249458
CYP19A1	31	404.07697	0.517094
FLT3	31	46.140938	0.5031185
CXCR2	31	175.52473	0.4979424
ITGB2	30	148.1003	0.50627613
COL1A1	29	296.9411	0.5073375
ALOX5	29	185.88608	0.4979424
MME	29	144.7264	0.5031185
SELE	29	28.388603	0.5073375
MAOB	29	515.8816	0.5010352
TYK2	27	24.926287	0.4888889
C5AR1	27	228.43706	0.49488753
FABP4	27	190.58704	0.48692152
DRD2	27	335.46796	0.48015872
ADAM17	27	73.78157	0.50840336
CTSD	27	128.18352	0.48790324
NR1H4	27	145.59619	0.48790324
SCD	26	126.67601	0.48594376
CYSLTR1	26	269.9115	0.484
PTPN1	26	99.50714	0.4979424
CPT1A	25	179.46625	0.48594376
PRKDC	25	77.342224	0.45833334
BRD4	25	107.75064	0.47920793

MAPK9	25	169.27304	0.49488753
EPHX2	25	384.53644	0.49590164
NOS1	25	82.30492	0.5052192
SREBF2	24	90.02289	0.484
TRPV1	24	276.70435	0.50416666
JAK3	24	14.101283	0.48207173
CYP2C19	24	251.26617	0.47265625
FABP1	23	97.72358	0.49488753
PTPN6	23	11.335422	0.46183205
HDAC3	23	81.28953	0.48207173
SLC6A4	23	260.09894	0.48790324
TEK	22	13.755866	0.47173488
LPAR1	22	110.351395	0.5010352
NR3C2	22	100.29859	0.484
ACHE	22	259.17264	0.5010352
EDNRA	22	90.91481	0.4908722
BDKRB1	22	163.26648	0.46360153
SHH	22	18.599031	0.4908722
CTSK	21	56.45798	0.4908722
PTGER4	21	73.82319	0.4928717
IDO1	21	34.3313	0.4928717
CHRM2	21	382.38364	0.48987854
CTSG	21	23.181784	0.46360153
PSEN1	21	193.61758	0.4811133
CYP27B1	20	95.91659	0.49590164
VCP	20	122.27083	0.45746693
CNR1	20	122.64809	0.49488753
MMP13	20	8.280192	0.48015872
HTR1A	20	122.3173	0.45746693
GSR	20	183.27068	0.49387756
MMP12	19	27.160746	0.47173488
GLP1R	19	98.069496	0.4908722
CTSL	19	10.220141	0.47081712
FABP3	18	94.29327	0.45746693
NR1H3	18	22.327774	0.4680851
NAMPT	18	26.790113	0.48207173
CSK	18	18.35728	0.46360153
CYP17A1	18	63.150826	0.45574388
LRRK2	18	87.635185	0.4699029
PTGES	18	53.34522	0.4699029
PLA2G2A	18	61.129173	0.4811133
SPHK1	18	67.61319	0.49488753
PTGER3	17	79.31283	0.47265625
PFKFB3	17	245.9484	0.46899223

ALOX15	17	31.020771	0.4680851
LTB4R	17	78.05075	0.47544205
SLC6A3	17	81.415085	0.41438356
EDNRB	17	46.581528	0.4653846
BCHE	17	132.84402	0.4888889
MAP3K7	16	58.74407	0.46095237
CPT2	16	45.043716	0.4368231
PTAFR	16	29.349619	0.46718147
G6PD	16	137.17981	0.46899223
ALOX5AP	16	116.13854	0.44567218
FLT4	15	3.3704803	0.4699029
S1PR1	15	22.430683	0.47544205
HSD11B1	15	65.72465	0.46360153
FKBP1A	15	162.78792	0.46718147
LPAR3	15	48.197487	0.46007603
TRPA1	15	67.25021	0.47173488
GHSR	15	75.45763	0.46095237
CHRM1	15	100.66962	0.42756185
CDK5	15	47.166607	0.45574388
FAAH	15	83.25942	0.45833334
SERPINA6	15	63.468643	0.4384058
CMA1	15	8.39869	0.46449137
MIF	15	14.152601	0.47358122
LTA4H	15	41.303024	0.46007603
FDFT1	14	37.639435	0.42605633
TAB1	14	7.4613657	0.47920793
ALOX12	14	23.494371	0.42605633
GLUL	13	59.18289	0.44649446
CA9	13	180.57759	0.45746693
SHBG	13	21.054659	0.46271512
ITGAL	13	5.4499445	0.45403376
HRH1	13	42.233006	0.46628132
PDE5A	13	27.41314	0.44981414
HSD11B2	13	22.876257	0.41438356
ROCK2	12	6.6985397	0.4680851
SIGMAR1	12	39.114388	0.45149255
MAPKAPK2	12	4.0786886	0.46718147
AOC3	12	14.168615	0.45833334
HTR2B	12	33.559658	0.41724136
TBXA2R	12	33.15819	0.41016948
FABP2	12	134.24051	0.46007603
DRD4	12	39.513943	0.44160584
ADH1B	12	82.33011	0.4
NR1H2	11	1.629264	0.42605633

PRKAG1	11	26.947361	0.41367522
RBP4	11	21.630716	0.45149255
PRKAA2	11	28.524239	0.4352518
AVPR2	11	38.16219	0.45746693
PSEN2	11	39.312073	0.41652325
OPRD1	11	50.718716	0.45574388
PTGIR	10	26.1019	0.43603605
ENPP2	10	25.02456	0.4440367
CPT1B	10	0	0.4186851
GPBAR1	10	27.740772	0.47173488
MC4R	10	44.98983	0.45403376
ATR	10	11.053308	0.39349595
CDK9	10	4.136993	0.41724136
SOAT1	10	10.106675	0.41652325
PLA2G5	10	4.6379395	0.430605
CYP11B1	10	6.3251243	0.40266222
PTGFR	10	37.738903	0.42233858
CYP11B2	10	6.4663267	0.40468228
ENPEP	10	3.9623773	0.4440367
EPHX1	10	68.09821	0.4074074
PRKAB1	9	6.5417204	0.40066224
PTGER2	9	10.632319	0.4368231
GYS1	9	28.118826	0.38969404
GABRA1	9	27.106707	0.41509435
CA2	9	108.10967	0.4352518
ADRA2B	9	61.802406	0.42756185
ADRA1A	9	23.022242	0.40199336
KCNJ11	9	179.03975	0.4216028
ABCC1	9	57.62285	0.43214285
NPC1L1	8	3.3503332	0.43137255
RARB	8	4.0730124	0.4489796
EGLN1	8	10.558184	0.45233646
F7	8	83.40343	0.44160584
SLC5A1	8	9.39466	0.4344704
TOP1	8	7.7753015	0.40468228
LIPA	7	3.4532475	0.417962
RORA	7	20.246744	0.43603605
CFD	7	0.4759548	0.44485295
F10	7	52.78826	0.43137255
RORC	7	18.766493	0.43137255
PDE4D	7	57.3904	0.41941074
CHRM3	7	9.243404	0.3872
PDE4A	6	21.385345	0.4352518
CNR2	6	6.226933	0.41226575

PDE4B	6	47.09619	0.44160584
PIM1	6	12.921712	0.40066224
CACNA2D1	6	28.157463	0.36065573
DUSP16	5	0	0.39933994
TACR2	5	2.6915865	0.39413682
ADH1C	5	11.615835	0.3628186
ACP1	5	4.8987827	0.41580757
SLC22A5	5	9.4700985	0.38110235
ABCC9	5	23.47564	0.36445785
SLC16A1	4	13.415223	0.34920636
DYRK1A	4	10.701817	0.38969404
PRKCH	4	5.293567	0.36722305
F9	3	0	0.34472933
RAMP1	3	3.1272988	0.4
CHRNA7	3	0.9697067	0.36666667
ICMT	2	0.14139344	0.38110235
CALCRL	2	0.11	0.39413682
ADORA3	2	0	0.39933994
PI4KB	1	0	0.35021707

Supplementary Table S2. Binding Energies from Molecular Docking Between Core Target Proteins and Active Components

	Hexadecanoic acid	Heptadecanoic acid	9,12-Octadecadienoic acid (Z, Z)-	9-Octadecenoic acid	Octadecanoic acid	9,11-Octadecadienoic acid,	Octadecanoic acid, 9,10,12-trimethoxy-	11-Eicosenoic acid	Eicosanoic acid
IL6	-3.73±0.12	-4.37±0.29	-4.83±0.12	-4.37±0.15	-4.33±0.06	-5±0.17	-4.8±0.1	-4.27±0.12	-3.73±0.06
TNF	-3.4±0.17	-4.27±0.15	-4.13±0.15	-4.67±0.21	-3.9±0	-4.5±0	-4.8±0.17	-3.8±0	-4.1±0.1
SRC	-2.9±0.2	-3.3±0	-3.83±0.06	-4.07±0.06	-3.4±0.17	-3.73±0.15	-4.47±0.12	-3.4±0.1	-3.4±0
PPAGR	-4±0	-5.27±0.12	-5.23±0.25	-5.07±0.25	-5.37±0.15	-5±0.2	-5.5±0	-5.23±0.15	-4.67±0.06
PTGS2	-3.37±0.25	-6.77±0.12	-4.93±0.25	-4.8±0.1	-6.83±0.25	-4.8±0.17	-6.47±0.21	-4.77±0.12	-4.7±0.1

MAPK3	-4.87±0.42	-4.9±0.26	-6.87±0.12	-6.87±0.12	-6.7±0	-5.5±0.1	-6.5±0.1	-6.43±0.32	-4.87±0.06
BCL2	-3.37±0.15	-4.37±0.12	-4.47±0.15	-4.83±0.06	-4.33±0.06	-4.93±0.15	-5±0.1	-4.37±0.15	-4.13±0.15
EGFR	-3.63±0.12	-4.47±0.23	-5.07±0.12	-4.67±0.12	-4.53±0.35	-4.5±0.35	-5.27±0.23	-4.73±0.15	-4.27±0.12
IL10	-2.87±0.21	-4.73±0.23	-5.03±0.15	-4.83±0.12	-4.41±0.09	-5.3±0.1	-5.13±0.06	-5±0.26	-5.17±0.06
MMP9	-3.67±0.25	-3.67±0.06	-6.47±0.15	-5.67±0.46	-5.47±0.06	-4.7±0.1	-7.07±0.23	-5.57±0.4	-5.93±0.15

Supplementary Table S3. Detailed Information of Western Blot Antibodies

target protein	vendor	catalog number	dilution used	host species
Vimentin	proteintech	10366-1-AP	1:10000	Rabbit
TGF beta1	AFFINITY	AF1027	1:2000	Rabbit
Alpha-SMA	AFFINITY	AF1032	1:1000	Rabbit
Fibronectin	proteintech	15613-1-AP	1:20000	Rabbit
Collagen Type I	proteintech	14695-1-AP	1:4000	Rabbit
Collagen Type III	proteintech	22734-1-AP	1:1000	Rabbit
PI3Kinase	Bioss	bsm-52218R	1:2000	Rabbit
Phospho-PI3Kinase	Bioss	bs-6417R	1:2000	Rabbit
AKT	proteintech	10176-2-AP	1:1000	Rabbit
phospho-AKT	Bioss	bsm-60645R	1:2000	Rabbit

Supplementary Table S4.Extracted Ion Chromatograms for all nine identified fatty acid methyl esters

Ingredient	Ion Chromatograms
Hexadecanoic acid, methyl ester	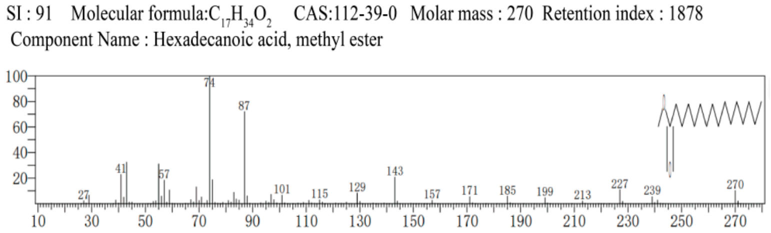
Heptadecanoic acid, methyl ester	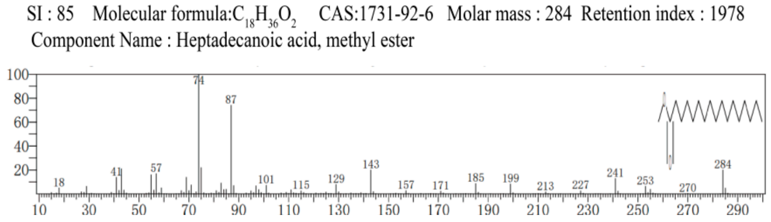
9,12-Octadecadienoic acid (Z, Z)-, methyl ester	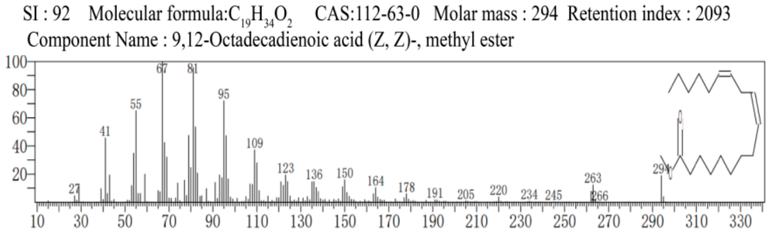
9-Octadecenoic acid, methyl ester, (E)-	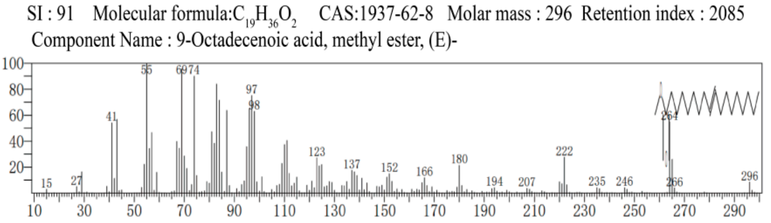
Octadecanoic acid, methyl ester	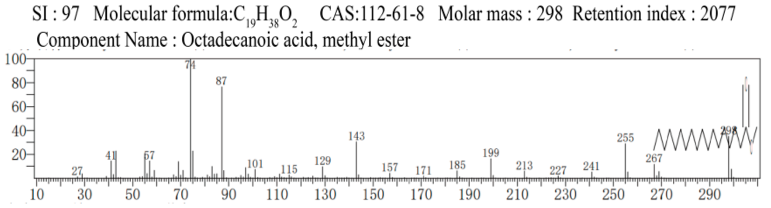
9,11-Octadecadienoic acid, methyl ester, (E, E)-	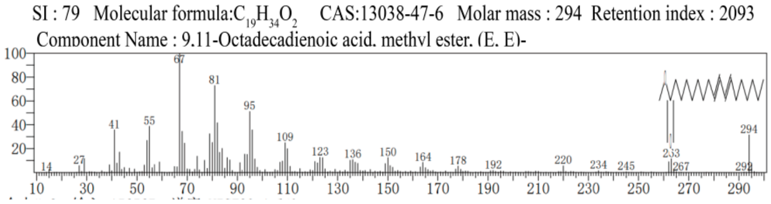

Octadecanoic acid, 9,10,12-trimethoxy-, methyl ester

SI : 76 Molecular formula:$C_{22}H_{44}O_5$ CAS:55255-75-9 Molar mass : 388 Retention index : 2411

Component Name : Octadecanoic acid, 9,10,12-trimethoxy-, methyl ester

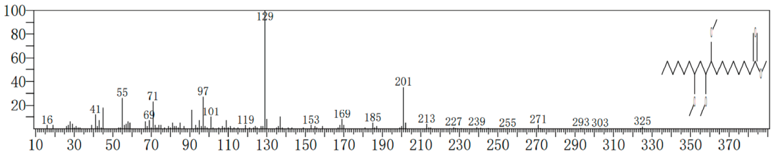

11-Eicosenoic acid, methyl ester

SI : 81 Molecular formula:$C_{21}H_{40}O_2$ CAS:3946-08-5 Molar mass : 324 Retention index : 2284

Component Name : 11-Eicosenoic acid, methyl ester

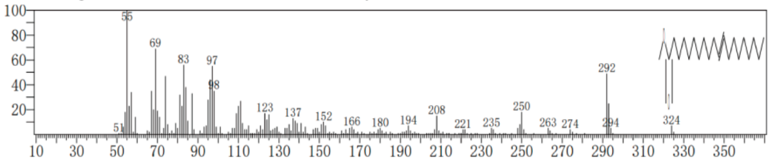

Eicosanoic acid, methyl ester

SI : 90 Molecular formula:$C_{21}H_{42}O_2$ CAS:1120-28-1 Molar mass : 388 Retention index : 2276

Component Name : Eicosanoic acid, methyl ester

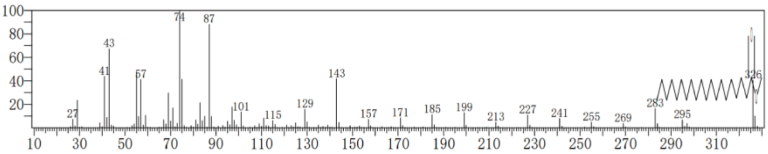

Supplement: Supplementary file 1 [file foods-15-01182-s001.zip › foods-4134605-supplementary.pdf]
